# Multimodal lifestyle engagement patterns support cognitive stability beyond neuropathological burden

**DOI:** 10.1186/s13195-023-01365-9

**Published:** 2023-12-18

**Authors:** Emily W. Paolillo, Rowan Saloner, Anna VandeBunte, Shannon Lee, David A. Bennett, Kaitlin B. Casaletto

**Affiliations:** 1 Department of Neurology, Memory and Aging Center, Weill Institute for Neurosciences, University of California, San Francisco, CA 94158 USA; 2 Department of Neurological Sciences, Rush Medical College, Chicago, IL USA

**Keywords:** Aging, Dementia, Neuropsychology, Neuropathology, Exercise, Social activity, Sleep, Life space, Mild cognitive impairment, Latent profiles

## Abstract

**Background:**

Modifiable lifestyle behaviors account for a large proportion of dementia risk. However, the combined contributions of multidomain lifestyle patterns to cognitive aging are poorly understood, as most studies have examined individual lifestyle behaviors in isolation and without neuropathological characterization. This study examined data-driven patterns of lifestyle behaviors across multiple domains among older adults and tested their associations with disease-specific neuropathological burden and cognitive decline.

**Methods:**

Participants included 2059 older adults enrolled in the longitudinal Memory and Aging Project (MAP) at the Rush Alzheimer’s Disease Center; none of whom had dementia at baseline (73% no cognitive impairment (NCI), 27% mild cognitive impairment [MCI]). All participants completed cognitive testing annually. Lifestyle factors were measured during at least one visit and included (1) actigraphy-measured physical activity, as well as self-reported (2) sleep quality, (3) life space, (4) cognitive activities, (5) social activities, and (6) social network. A subset of participants (*n* = 791) had autopsy data for which burden of Alzheimer’s disease (AD), cerebrovascular disease (CVD), Lewy body disease, and hippocampal sclerosis/TDP-43 was measured. Latent profile analysis across all 2059 participants identified distinct subgroups (i.e., classes) of lifestyle patterns. Linear mixed-effects models examined relationships between lifestyle classes and global cognitive trajectories, with and without covarying for all neuropathologies. Classes were also compared on rates of incident MCI/dementia.

**Results:**

Five classes were identified: Class 1_Low Life Space_ (lowest lifestyle engagement), Class 2_PA_ (high physical activity), Class 3_Low Avg_ (low to average lifestyle engagement), Class 4_Balanced_ (high average lifestyle engagement), and Class 5_Social_ (large social network). Classes 4_Balanced_ and 5_Social_ had the lowest AD burden, and Class 2_PA_ had the lowest CVD burden. Classes 2–5 had significantly less steep global cognitive decline compared to Class 1_Low Life Space_, with comparable effect sizes before and after covarying for neuropathological burden. Classes 4_Balanced_ and 5_Social_ exhibited the lowest rates of incident MCI/dementia.

**Conclusions:**

Lifestyle behavior patterns among older adults account for differential rates of cognitive decline and clinical progression. Those with at least average engagement across all lifestyle domains exhibit greater cognitive stability after adjustment for neuropathology, highlighting the importance of engagement in multiple healthy lifestyle behaviors for later life cognitive health.

**Supplementary Information:**

The online version contains supplementary material available at 10.1186/s13195-023-01365-9.

## Introduction

The prevalence of dementia is precipitously rising, yet development of cognitive impairment with age is not inevitable. Converging lines of evidence suggest that over 40% of dementia cases are attributable to modifiable exposures and lifestyle factors [1]. Optimal engagement in later life behaviors such as physical activity, sleep, and cognitive and social activities has each been linked with reduced cognitive decline, even among older adults with autosomal dominant genetic risk for dementia. These data underscore a key need to develop primary prevention approaches for brain health. Yet, despite significant advancements in our understanding of individual dementia prevention techniques, lifestyle behaviors do not occur in isolation, and holistic understanding of lifestyle patterns for dementia prevention has lagged.

Indeed, the vast majority of studies have examined potential neuroprotective behaviors one at a time, despite the high level of interconnection across such lifestyle factors. Therefore, little is known about whether specific neuroprotective patterns of lifestyle behaviors exist [2–4]. Previous work from Carlson and colleagues [5] showed that participation in a *variety* of lifestyle behaviors is most associated with reduced risk of cognitive aging or the development of dementia. Similarly, data from the randomized controlled FINGER trial demonstrate that a multimodal approach to lifestyle optimization can support cognitive health over a 2-year period in older adults at risk for dementia [6]. These and other studies suggest that a deeper understanding of multimodal lifestyle patterns for dementia prevention is highly warranted. However, most studies in this area do not account for interrelationships among multiple lifestyle factors [7, 8] or binarize (yes/no) these variables [9, 10], which may not fully capture the natural spectrum of behavior. Examining a wide range of lifestyle factors in combination is both ecologically valid and may provide additional insights into patterns that most strongly relate to better brain and cognitive outcomes.

Another limitation of the existing literature examining lifestyle behaviors and brain health is the lack of contextual neuropathological information available. For instance, animal models and some emerging human data indicate that physical activity and sleep may directly contribute to pathological burden as well as impact one’s ability to engage in neuroprotective behaviors [11–14]. These data suggest that understanding the neuropathological milieu may be highly relevant when disentangling the effects of lifestyle on cognitive health. Notably, of the studies that have examined lifestyle factors and resistance to pathological change (e.g., Alzheimer’s disease pathology), few have incorporated multiple facets of lifestyle [10, 11]. Understanding how modifiable risk factors associate with risk of specific pathology development and incrementally relate to cognitive health beyond neuropathological burden (e.g., disease stage) is important to inform person- and disease-specific brain health recommendations.

To better understand the combined contribution of multiple, modifiable lifestyle patterns on neuropathology burden and cognitive aging, we leveraged multimodal lifestyle and longitudinal cognitive data from adults without baseline dementia enrolled in the Rush Memory and Aging Project (MAP) neuropathology cohort. Prior work in this cohort has reported independent relationships among individual lifestyle factors (e.g., cognitive activities, physical activity, social engagement), cognitive change, and individual neuropathologies (e.g., AD burden) [11, 15, 16]. Here, we extend that work. We employed latent profile analysis to empirically derive subgroups of participants with similar behavior patterns across different lifestyle domains, including physical activity, cognitive activity, social engagement, sleep, and environmental enrichment. We examined the relationship between lifestyle patterns and neuropathology burden as well as longitudinal cognitive trajectories and rates of incident CI. Systematic model comparisons determined whether lifestyle pattern effects on cognition were comparable to, and independent of, the relative effects of disease burden across multiple neuropathologies.

## Methods

### Participants

Study participants were more than 2000 older adults enrolled in the longitudinal MAP cohort [17]. Participants completed cognitive testing and received clinical diagnoses at baseline and each annual follow-up visit. For the present analysis, we included individuals who were free of dementia at baseline (i.e., cognitively unimpaired and mild cognitive impairment [MCI] only) and had available data for at least half of the six lifestyle measures of interest (see below). Of the 2059 study participants, 791 had available autopsy data. The MAP study was approved by a Rush University Medical Center Institutional Review Board, conducted in accordance with the latest Declaration of Helsinki. Participants provided written informed and repository consents and Anatomic Gift Act for organ donation.

### Lifestyle measures

#### Physical activity

Omnidirectional actigraphy monitors were worn by participants on the nondominant wrist and measured rest/activity continuously for 24 h a day, for up to 10 days per visit (Actical; Mini Mitter). Activity counts were extracted in 15-s epoch estimates, provided by Actical. Incomplete data were detected via inspection of accelerometer recordings through an automated program that flagged average daily counts at extreme levels: ≈ 0/day or > 500/day. Only participants with valid data for 1+ days were included in analyses. Daily physical activity values included a summary of both exercise and non-exercise activities and was calculated as the average sum of all 15-s epoch daily activity counts for all full days of actigraphy data.

#### Sleep quality

Sleep quality was measured using a modified version of the Pittsburg Sleep Quality Index (PSQI) and select items from the Berlin Questionnaire, per previous protocols [18, 19]. Individual questionnaire items were aggregated to calculate scores for the following six PSQI components: sleep latency, sleep duration, sleep efficiency, sleep disturbances, usage of sleeping medications, and daytime dysfunction. These sleep component scores were summed and reverse scored to assign a total sleep quality score ranging from 0 to 16, with higher indicating better sleep quality.

#### Social activities

Participant social activity was assessed as frequency of engagement in social activity in late life [20]. On a 6-item survey, participants were asked to rate how often they engaged in common types of activities involving social interaction on a 5-point scale (e.g., 1 = once a year, 2 = several times a year, 3 = several times a month, 4 = several times a week, 5 = every day or almost every day). Higher values indicate more frequent participation in the listed activity. The social activity composite measure was calculated by averaging all item scores, with higher scores representing greater social activity (range 1 to 5).

#### Social network

Social network was assessed as a measure of network size [15] and was quantified based on standardized questions about the following: (1) number of close family members and friends that were seen by the participant and (2) how often the participant interacted with them (a minimum of once a month). Specifically, social network size was quantified by the number of community members, relatives, and friends seen at least once a month (range 0 to no upper limit).

#### Life space

Life space has been defined as distance traveled from home in daily life and is posited to reflect degree of spatial movement through an individuals’ environment as a proxy for environmental enrichment. A modified version of the Life Space Questionnaire was used to develop a scale that quantifies spatial movement in up to six specific environment zones (bedroom, porch/patio, parking lot/yard, within neighborhood, outside neighborhood, outside of town) [21]. Participants reported their presence in each of the zones within the past week of questionnaire administration (yes = 1 or no = 0). Individual life space scores were calculated as a sum of all binary responses and ranged from 0 to 6, with higher scores indicating greater life space.

#### Cognitive activities

Cognitive activity was measured as frequency of participation in cognitively stimulating behaviors in late life [22]. The composite score was generated by averaging individual frequency scores across seven cognitively stimulating activities in the past year, including reading, writing letters, visiting a library, and playing games of skill (e.g., chess, checkers). These activities were specifically included to tap into skills involving information processing or retention and being relatively accessible (few barriers to participation). Each item was scored on a 5-point scale, with higher values indicating more frequent participation (range 1 to 5).

### Cognitive testing

A global cognitive composite score was derived from a battery of 21 cognitive tests administered to participants each year. Tests measured episodic memory, semantic processing, working memory, processing speed, and executive functioning. Raw test scores from the 19 tasks were first converted to z-scores and then averaged to produce a global cognitive function summary measure, as previously described [23]. Mean and standard deviation at baseline were used to compute the z-scores.

### Neuropathology

Brain autopsy procedures were completed by examiners blinded to all clinical information. Brains were removed, and hemispheres were cut coronally into 1-cm slabs using a Plexiglas jig, with one hemisphere preserved in 4% paraformaldehyde. Following gross examination of each hemisphere, nine brain regions of interest were dissected from the fixed tissue and processed and embedded in paraffin, including midfrontal, midtemporal, basal ganglia, thalamus, midbrain, inferior parietal, anterior cingulate, and entorhinal and hippocampal cortices. Paraffin blocks were then stained for assessment of pathology. Additional information on these procedures has been previously described in detail [24–27]. Neuropathologies were categorized into four groupings: Alzheimer’s disease, cerebrovascular disease, Lewy body disease, and hippocampal sclerosis/TDP-43.

#### Alzheimer’s disease

A measure of global Alzheimer’s disease (AD) pathology burden was created using a quantitative summary of AD pathology, based on methods previously described [17]. AD pathology involved total count of neurofibrillary tangles, neuritic plaques, and diffuse plaques. Regional counts were derived from 15 regions (e.g., hippocampus and the midfrontal, midtemporal, inferior parietal, and entorhinal cortices) and scaled by dividing by the corresponding regional standard deviation. Each of the scaled regional measures were then averaged into three summary pathology measures (neurofibrillary tangles, neuritic plaques, and diffuse plaques); finally, the three summary pathology measures were then averaged into a global AD pathology metric, following previous approaches [17].

#### Cerebrovascular disease (CVD)

Cerebral amyloid angiopathy (CAA) was assessed by examining amyloid deposition in meningeal and parenchymal vessels using a previously described protocol [28]. CAA scores were classified into a severity rating (0 = none, 1 = mild, 2 = moderate, 3 = severe) using cutoffs determined by the neuropathologist [27].

Large vessel cerebral atherosclerosis ratings were completed through visual inspection of the circle of Willis at the base of the brain using methods previously described [29]. Severity was graded (0 to 6) based on the extent of involvement of each artery and number of arteries involved and was collapsed to 4 levels for analysis (0 = no significant atherosclerosis observed, 3 = atherosclerosis was examined in more than half of all visualized arteries, and/or more than 75% occlusion of one or more vessels).

Arteriolosclerosis was defined by any histological change found in the small vessels, including smooth muscle degeneration, fibrohyalinotic thickening of arterioles with consequent narrowing of the vascular lumen, and intimal deterioration. The vessels were evaluated in the anterior basal ganglia using a semiquantitative grading system that has been previously described elsewhere [30]. For the cerebrovascular composite, the levels were compressed into four levels (0 = none, 1 = mild, 2 = moderate, 3 = severe).

Gross infarcts were identified visually and confirmed histologically, while micro infarcts were identified under microscopy using hematoxylin and eosin (H&E) stain [24, 31]. Gross and micro infarcts were coded on a severity scale (0 = none present, 1 = one or more infarction, regardless of location) based on methods previously described [32, 33].

A cerebrovascular composite was created by taking the sum of CAA (0 to 3) + arteriolosclerosis (0 to 3) + atherosclerosis (0 to 3) + gross chronic infarcts (0 or 1) × 3 + micro infarcts (0 or 1) × 3. Each cerebrovascular pathology was weighted to be equally represented in the composite (range = 0–15) based on methods previously used for the creation of summary scores of cerebrovascular dysfunction [34–36].

#### Lewy body disease (LBD)

The presence of Lewy body pathology was determined using antibodies to α-synuclein [37]. LBD was binarized as no Lewy body pathology (0) or Lewy bodies present in nigral, limbic, or neocortical regions (1).

#### Hippocampal sclerosis (HS)/TDP-43

Hippocampal sclerosis was determined using H&E stain on a section of the mid-hippocampus, based on methods previously described elsewhere [25]. TDP-43 cytoplasmic inclusions in neurons and glia were determined for eight regions (yes vs. no), including amygdala, entorhinal cortex, hippocampus CA1, hippocampus dentate gyrus, and anterior temporal pole, midtemporal, orbital frontal, and midfrontal cortices. TDP-43 inclusions were determined using antibodies to phosphorylated TDP-43 (pS409/410; 1:100), and TDP-43 distribution was grouped into four stages (0: none present; 1: localized to amygdala; 2: extension to limbic regions; 3: extension to the neocortex). TDP-43 was considered present if positive for stages 2 or 3 [26, 38]. When examining the data, there was a high degree of overlap between participants with hippocampal sclerosis and TDP-43, such that only *n* = 7 demonstrated hippocampal sclerosis without TDP-43. Therefore, to reduce collinearity of pathologies represented, individuals with hippocampal sclerosis only were excluded, and participants were coded into the following three groups: no TDP-43 or hippocampal sclerosis (0), only TDP-43 (1), and both TDP-43 and hippocampal sclerosis present (2).

### Statistical analyses

Latent profile analysis (LPA) was used to identify homogeneous groups of participants (*n* = 2059) with similar lifestyle patterns based on the 6 available lifestyle measures described above: physical activity, sleep quality, life space, late life cognitive activities, late life social activities, and size of social network [39]. Raw scores for each lifestyle metric were averaged within persons across all available study visits to estimate stable, later life engagement in each lifestyle domain [40, 41]. This methodology was employed as repeated measurements provide a more reliable estimate of stable trends in behavior compared to single-timepoint measurements [42, 43]. To enhance interpretability, these averaged late life lifestyle scores were z-score transformed prior to inclusion in the LPA. Participants were required to have data for at least three of the six lifestyle metrics to be included. As part of standard LPA procedures, missing data were imputed using a random forest imputation algorithm from the MissForest package in R [44, 45]. To determine the optimal number of groups (i.e., classes) underlying the lifestyle data, separate models with an increasing number of latent classes were estimated and compared using the following model fit indices: log likelihood, Bayesian information criterion (BIC), Akaike information criterion (AIC), and the bootstrapped likelihood ratio test (BLRT). Model diagnostics are also reported (i.e., entropy and average posterior classification probability). For each model with *k* number of classes, the BLRT compared the log likelihood to that of the model with *k*-1 classes to examine whether adding the *k*th class significantly improved model fit [46]. In addition to examining fit indices, a qualitative examination of class size and interpretability was utilized to select the model with the number of classes best fitting the data. Participants are identified as belonging to one class. LPA was conducted using R version 4.2.0 and MPlus version 7.4.

To examine differences in demographics and clinical characteristics by latent lifestyle class membership, ANOVA or chi-square tests were used for continuous or categorical variables, respectively. ANOVAs were also used to examine how lifestyle classes directly relate to burden of each measured neuropathology. Next, we examined how lifestyle patterns associated with later life cognitive trajectories. Linear mixed-effects (LME) models were used to examine the interaction between latent lifestyle class membership and time on global cognition in a two-step fashion: (1) without covarying for the effects of neuropathology on cognitive trajectory and (2) covarying for neuropathology on cognitive trajectory. Both models included person-specific random intercepts and a random effect of time, as well as time-invariant covariates (i.e., baseline age, sex, education, and total number of study visits). Including these random effects in the model provides estimation of individual intercepts (i.e., levels of the global cognition at baseline) and individual slopes (i.e., trajectories of global cognition over time) for each person, which allows proper examination of whether class membership explains variance in these individual cognitive slopes. Estimates from LME regression models are reported as standardized betas (β), which describe the strength of the relationship between predictor and outcome in units of standard deviations. A post hoc LME model was conducted to examine the unique contribution of each individual lifestyle measure on global cognitive trajectory, covarying for neuropathology, age, and sex. Additionally, analyses examined rates of incident CI from first to last visit by lifestyle group using chi-square tests. FDR-adjusted *p*-values were utilized for pairwise class comparisons.

## Results

### Participant characteristics

Table 1 presents demographic, clinical, lifestyle, and neuropathological characteristics in the study sample. Participants were 75% female and on average 80 years old at baseline (range: 53–100) with 14.9 years of education. At baseline, 73% of participants were NCI, and 27% were diagnosed with MCI. At last study visit, 55% of participants were NCI, 24% MCI, and 21% dementia. Participants completed an average of seven annual cognitive assessments, and the average time from baseline visit to death in the autopsy subcohort was 7.6 years.

**Table 1:** Participant characteristics (*N* = 2059)

	**Mean (SD) or *N*** **(%)**	
*Demographics*		
Baseline age	79.75 (7.55)	
Education	14.91 (3.31)	
Sex (female)	1537 (75%)	
Race/ethnicity		
White	1922 (93%)	
Black/African American	109 (5%)	
Other	28 (2%)	
*Study characteristics*		
Number of visits	7.03 (4.69) [range = 1–22]	
Total time in study until death (*n = 791*)	7.61 (4.32)	
*Lifestyle characteristics*		
Average physical activity raw score	2.07 (1.15)	
Average sleep quality raw score	10.18 (2.46)	
Average life space raw score	5.15 (1.00)	
Average cognitive activities raw score	3.03 (0.64)	
Average social activities raw score	2.48 (0.53)	
Average social network raw score	6.66 (4.63)	
*Cognitive characteristics*		
Baseline global cognition	0.08 (0.55)	
Clinical diagnosis	Baseline	Last visit
Normal cognition	1510 (73%)	1130 (55%)
MCI	549 (27%)	492 (24%)
Dementia	-	437 (21%)
*Neuropathology (n = 791)*		
Alzheimer’s disease	0.71 (0.61)	
Cerebrovascular disease	5.46 (3.02)	
Lewy body disease (present)	176 (23%)	
Hippocampal sclerosis/TDP-43	0.61 (0.63)	

“Other” race/ethnicity includes the following: Asian, American Indian or Alaska Native, Native Hawaiian or Other Pacific Islander, other, or unknown

### Lifestyle profiles

Correlational analyses examining the six individual lifestyle measures (Table 2) evidenced relationships that ranged from minimal to medium (*r* range: 0.05 to 0.50). The majority of associations were statistically significant, with the exception of null associations between sleep and physical activity (*r* = 0.05), sleep and cognitive activities (*r* = 0.06), and physical activity and social network (*r* = 0.05).

**Table 2:** Pearson r correlation matrix of individual lifestyle factor Z-scores

	**PA**	**Sleep**^**a**^	**Life space**	**Cognitive activities**	**Social activities**	**Social network**
Physical activity	-					
Sleep^a^	0.05	-				
Life space	0.26***	0.11***	-			
Cognitive activities	0.10***	0.06	0.31***	-		
Social activities	0.20***	0.10**	0.50***	0.41***	-	
Social network	0.05	0.08*	0.24***	0.18***	0.38***	-

**p < .01. ***p < .001. ^a^Higher scores = better sleep quality

Table 3 displays model fit indices and diagnostics for LPA models with increasing number of classes (ranging from 1 to 6 classes). A five-class solution was selected as the optimal model on the basis of fit indices showing lower log likelihood, AIC, and BIC compared to lower-class solutions (BLRT *p*-value < 0.001) while maintaining optimal diagnostics (entropy and minimum average posterior classification probability > 0.80). Although a six-class solution yielded lower AIC and BIC values than the five-class solution, class profiles were less interpretable and had inferior model diagnostics. Given that lifestyle metrics were averaged across all study visits, which included follow-up visits in which participants had progressed to dementia, we performed a sensitivity analysis in which the computation of average lifestyle metrics was restricted to non-dementia timepoints (i.e., NCI, MCI). This analysis again yielded an optimal five-class solution with near identical class separation (entropy = 0.90) and lifestyle differences across class, thereby supporting stability of class profiles as independent of dementia state (e.g., rather than a consequence of dementia-related behavioral change).

**Table 3:** LPA fit indices and model diagnostics for class solutions 1 through 6

**Classes**	**Log likelihood**	**AIC**	**BIC**	**Smallest class size (% of sample)**	**BLRT**	**BLRT** ***p*-value**	**Entropy**	**Minimum average posterior probability**^**b**^
1	−16,573.15	33,170.30	33,237.86	2059 (100%)	-	-	1.00	1.00
2	−15,738.43	31,514.85	31,621.82	383 (18.6%)	1669.45	< .001	0.90	0.92
3	−15,473.25	30,998.49	31,144.87	100 (4.9%)	530.36	< .001	0.90	0.79
4	−15,252.65	30,571.30	30,757.09	91 (4.4%)	441.19	< .001	0.89	0.84
5^a^	−15,094.37	30,268.74	30,493.94	64 (3.1%)	316.57	< .001	0.89	0.84
6	−14,978.71	30,051.43	30,316.03	62 (3.0%)	231.31	< .001	0.78	0.80

^a^Selected as the most optimal class solution based on a combination of fit indices. ^b^Represents the minimal of the diagonal of the average latent class probabilities for most likely class membership

**Fig. 1:**
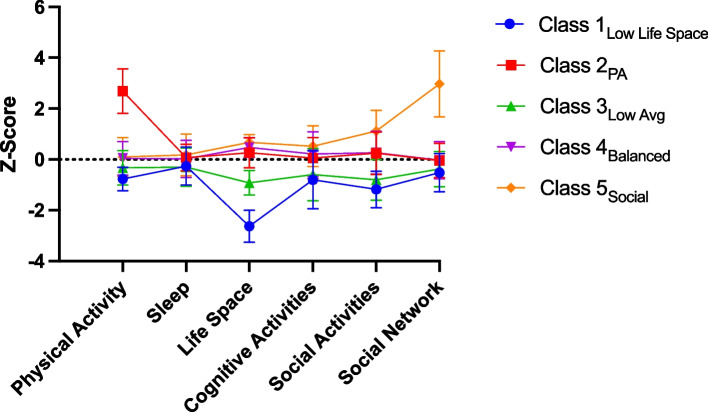
Five-class LPA solution. Colored lines represent average z-scores for each lifestyle activity across each class, with error bars representing standard deviations

As shown in Fig. 1, the five data-driven lifestyle classes that emerged can be described based on visual inspection of relative levels and peaks of engagement in each lifestyle domain. Class 1_Low Life Space_ had the lowest levels across all six measures, with particularly low levels of life space (*N* = 138). Class 2_PA_ demonstrated very high physical activity (PA) with average to high levels across the remaining 5 measures (*N* = 64). Class 3_Low Avg_ demonstrated low to average (avg) levels across all 6 measures (*N* = 394). Class 4_Balanced_ had average to high levels across all 6 measures (*N* = 1374). Finally, Class 5_Social_ had the largest social network, with high levels of social activities, cognitive activities, and life space, with average levels across the remaining 2 lifestyle measures (*N* = 89).

### Lifestyle class characteristics

Table 4 presents participant characteristics by lifestyle pattern class. Demographic differences by class were observed for age (mean age range: 76.0 years [Class 2_PA_] to 84.8 years [Class 1_Low Life Space_]), education (mean education range: 13.6 years [Class 1_Low Life Space_] to 15.6 years [Class 5_Social_]), and, to a lesser extent, sex [%female range: 65% [Class 5_Social_] to 81% [Class 2_PA_]). With regard to neuropathology, omnibus class differences were observed for AD and CVD, but not LBD or HS/TDP-43. Classes 4_Balanced_ and 5_Social_ exhibited the lowest levels of AD pathological burden, with pairwise comparisons showing that Class 4_Balanced_ had significantly lower AD burden than Classes 2_PA_ (*p* = .042) and 3_Low Avg_ (*p* = .006). In contrast, Class 2_PA_ exhibited the lowest CVD burden, with pairwise differences that were significant or approached significance compared to the other 5 classes (*p* range: < .001 to .061).

**Table 4:** Participant characteristics by class

	Class 1_Low Life Space_ (*n* = 138)	Class 2_PA_ (*n* = 64)	Class 3_Low Avg_ (*n* = 394)	Class 4_Balanced_ (*n* = 1374)	Class 5_Social_ (*n* = 89)	*p*-value
*Demographics*						
Baseline age	84.81 (6.53)	75.98 (7.61)	82.34 (6.91)	78.74 (7.47)	78.70 (6.60)	< 0.001
Education	13.56 (3.36)	14.22 (2.96)	13.89 (3.16)	15.33 (3.28)	15.60 (2.95)	< 0.001
Sex (female)	107 (78%)	52 (81%)	311 (79%)	1009 (73%)	58 (65%)	0.024
Race/ethnicity						0.224
White	134 (97%)	55 (86%)	364 (92%)	1284 (93%)	85 (96%)	
Black/African American	3 (2%)	9 (14%)	26 (6%)	68 (5%)	3 (3%)	
Other	1 (1%)	0 (0%)	4 (1%)	22 (2%)	1 (1%)	
*Study characteristics*						
Number of visits	5.59 (3.84)	8.28 (4.08)	7.52 (4.40)	7.01 (4.83)	6.47 (4.99)	< 0.001
*Lifestyle Characteristics*						
Average physical activity z score	−0.77 (0.46)	2.69 (0.88)	−0.33 (0.68)	0.04 (0.66)	0.09 (0.77)	< 0.001
Average sleep quality z score	−0.26 (0.75)	0.07 (0.52)	−0.30 (0.75)	0.02 (0.73)	0.18 (0.82)	< 0.001
Average life space z score	−2.63 (0.63)	0.27 (0.59)	−0.92 (0.48)	0.47 (0.39)	0.67 (0.31)	< 0.001
Average cognitive activities z score	−0.80 (1.14)	0.06 (0.81)	−0.59 (1.03)	0.21 (0.87)	0.52 (0.80)	< 0.001
Average social activities z score	−1.18 (0.72)	0.25 (0.84)	−0.80 (0.80)	0.27 (0.84)	1.11 (0.82)	< 0.001
Average social network z score	−0.52 (0.75)	−0.04 (0.68)	−0.38 (0.69)	−0.03 (0.73)	2.97 (1.30)	< 0.001
*Cognitive characteristics*						
Baseline global cognition	−0.36 (0.54)	0.10 (0.59)	−0.17 (0.53)	0.18 (0.52)	0.25 (0.47)	< 0.001
Baseline clinical diagnosis (normal)	70 (51%)	51 (80%)	254 (65%)	1060 (77%)	75 (84%)	< 0.001
Change in clinical diagnosis from baseline to last visit						< 0.001
Normal to normal	32 (23%)	35 (55%)	109 (28%)	782 (57%)	60 (67%)	
Normal to MCI	11 (8%)	9 (14%)	61 (16%)	158 (12%)	10 (11%)	
Normal to dementia	27 (20%)	7 (11%)	84 (21%)	120 (9%)	5 (6%)	
MCI to normal	3 (2%)	4 (6%)	19 (5%)	84 (6%)	2 (2%)	
MCI to MCI	29 (21%)	3 (5%)	51 (13%)	150 (11%)	10 (11%)	
MCI to dementia	36 (26%)	6 (9%)	70 (18%)	80 (6%)	2 (2%)	
*Neuropathology (n = 791)*						
Alzheimer’s disease	0.71 (0.63)	0.94 (0.70)	0.80 (0.62)	0.66 (0.58)	0.64 (0.57)	0.029
Cerebrovascular disease	6.63 (3.26)	3.76 (2.26)	5.84 (2.91)	5.10 (2.98)	5.50 (2.31)	< 0.001
Lewy body disease (present)	18 (20%)	5 (25%)	48 (22%)	100 (24%)	5 (26%)	0.951
Hippocampal sclerosis/TDP-43	0.63 (0.66)	0.65 (0.59)	0.66 (0.67)	0.57 (0.61)	0.53 (0.51)	0.545

### Lifestyle classes and cognitive trajectories

**Fig. 2:**
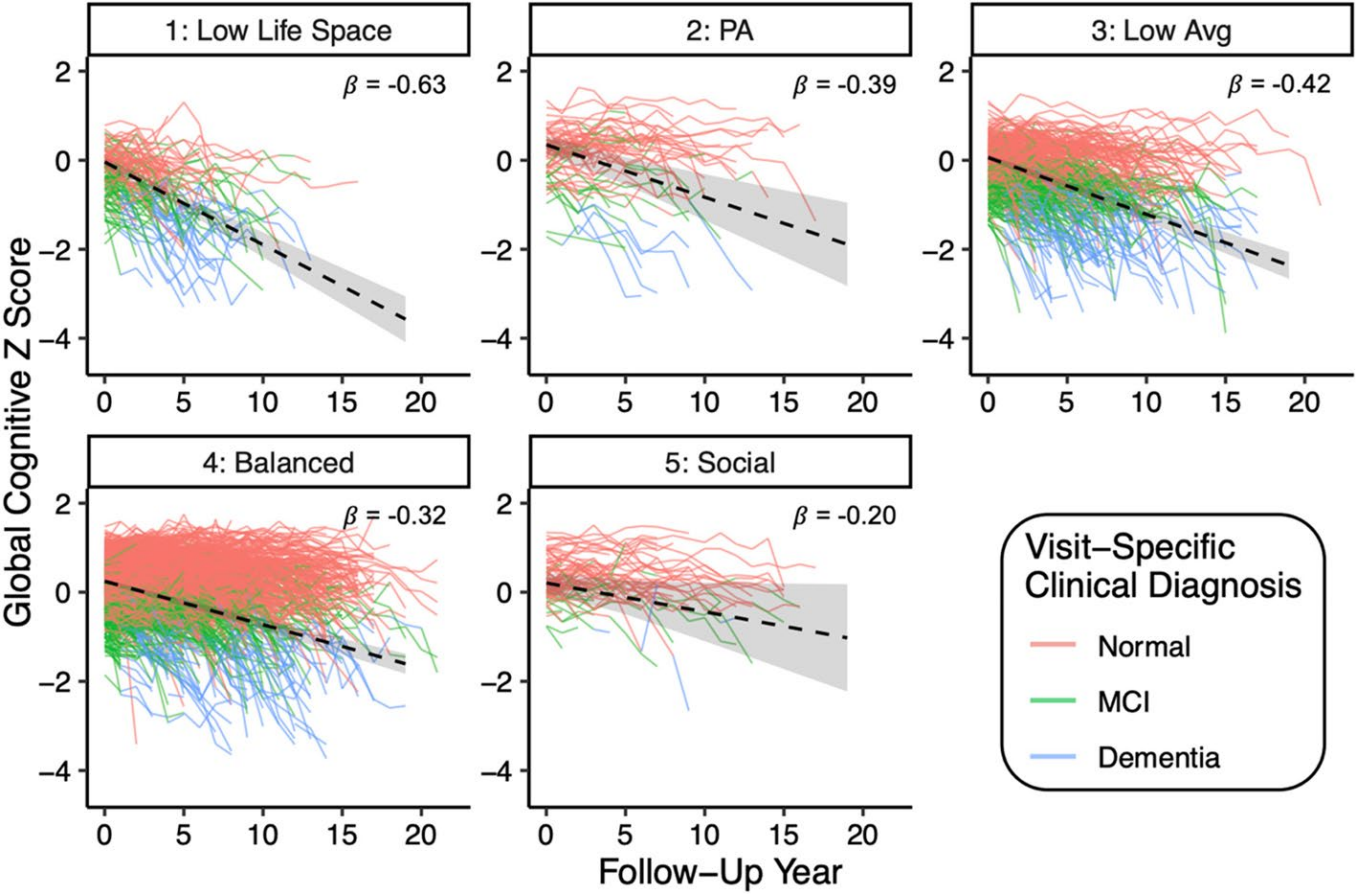
Spaghetti plot showing raw data for each participant at every visit by LPA-derived lifestyle class. Each colored line represents one participant. Colors represent participants’ clinical consensus diagnosis at each visit. Black dashed lines are estimated class-specific global cognitive trajectories from linear mixed effects model results (adjusting for pathology), shown with corresponding standardized slope estimates

A two-step linear-mixed effects regression model tested the association between lifestyle class and cognitive trajectories, before and after adjustment for neuropathological burden among the subset of 704 participants with autopsy data (Table 5). In Model 1, Classes 2–5 exhibited significantly less steep global cognitive decline (all time × class interaction *p*s < .05; Fig. 2) compared to reference Class 1_Low Life Space_ (time slope: *β* = −0.67, *p* < .001). Between Classes 2–5, Class 5_Social_ exhibited the flattest cognitive trajectory (time slope: *β* = −0.18), followed by Class 4_Balanced_ (time slope: *β* = −0.33), Class 2_PA_ (time slope: *β* = −0.45), and then Class 3_Low Avg_ (time slope: *β* = −0.49). In Model 2, AD, CVD, LBD, and HS/TDP-43 were added as independent pathological predictors of cognitive trajectories. AD, LBD, and HS/TDP-43 were each associated with significantly steeper cognitive decline (*p*s < .002), with AD showing the strongest effects compared to that of other pathologies, consistent with previous publications in this cohort [47]. In this pathology-adjusted model, Classes 2–5 continued to show slower cognitive decline compared to Class 1_Low Life Space_, with comparable effect sizes to Model 1 (Table 5). Further examination of pairwise class contrasts showed no statistically significant differences between Classes 2_PA_, 4_Balanced_, and 5_Social_ on cognitive trajectories in either Model 1 or Model 2. In addition, Model 1 results in this subset of 704 participants with autopsy data were comparable to that of the entire 2059 participant sample (Supplementary Table 1).

**Table 5:** Linear mixed-effects model results

	Model 1			Model 2		
	Std. beta	95% *CI*	*p*-value	Std. beta	95% *CI*	*p*-value
Baseline age	−0.08	−0.12, −0.05	< 0.001	−0.06	−0.10, −0.03	0.001
Sex (ref.: female)	−0.14	−0.22, −0.06	0.001	−0.13	−0.21, −0.05	0.001
Education	0.14	0.11, 0.18	< 0.001	0.14	0.10, 0.17	< 0.001
Total study visits	0.15	0.12, 0.19	< 0.001	0.18	0.14, 0.22	< 0.001
Time	−0.67	−0.78, −0.57	< 0.001	−0.63	−0.73, −0.54	< 0.001
Class 2_PA_ (ref.: 1_Low Life Space_)	0.64	0.29, 0.99	< 0.001	0.67	0.35, 0.99	< 0.001
Class 3_Low Avg_ (ref.: 1_Low Life Space_)	0.31	0.13, 0.50	0.001	0.35	0.18, 0.52	< 0.001
Class 4_Balanced_ (ref.: 1_Low Life Space_)	0.70	0.52, 0.87	< 0.001	0.66	0.50, 0.82	< 0.001
Class 5_Social_ (ref.: 1_Low Life Space_)	0.85	0.43, 1.26	< 0.001	0.76	0.39, 1.13	< 0.001
Time × Class 2_PA_	0.22	0.00, 0.44	0.049	0.24	0.04, 0.44	0.018
Time × Class 3_Low Avg_	0.18	0.06, 0.30	0.004	0.21	0.10, 0.32	< 0.001
Time × Class 4_Balanced_	0.34	0.22, 0.45	< 0.001	0.31	0.21, 0.42	< 0.001
Time × Class 5_Social_	0.49	0.22, 0.77	< 0.001	0.43	0.19, 0.67	0.001
AD	-	-	-	−0.22	−0.27, −0.17	< 0.001
CVD	-	-	-	−0.07	−0.12, −0.02	0.004
LBD (ref.: no.)	-	-	-	−0.27	−0.39, −0.16	< 0.001
HS/TDP-43	-	-	-	−0.11	−0.16, −0.06	< 0.001
Time × AD	-	-	-	−0.14	−0.17, −0.11	< 0.001
Time × CVD	-	-	-	−0.03	−0.06, 0.00	0.085
Time × LBD	-	-	-	−0.12	−0.19, −0.05	0.002
Time × HS/TDP-43	-	-	-	−0.05	−0.08, −0.02	0.001

Both model 1 and model 2 include 704 participants with complete neuropathology data (*N* = 87 in Class 1_Low Life Space_; *N* = 19 in Class 2_PA_; *N* = 202 in Class 3_Low Avg_; *N* = 380 in Class 4_Balanced_; *N* = 16 in Class 5_Social_)

**Fig. 3:**
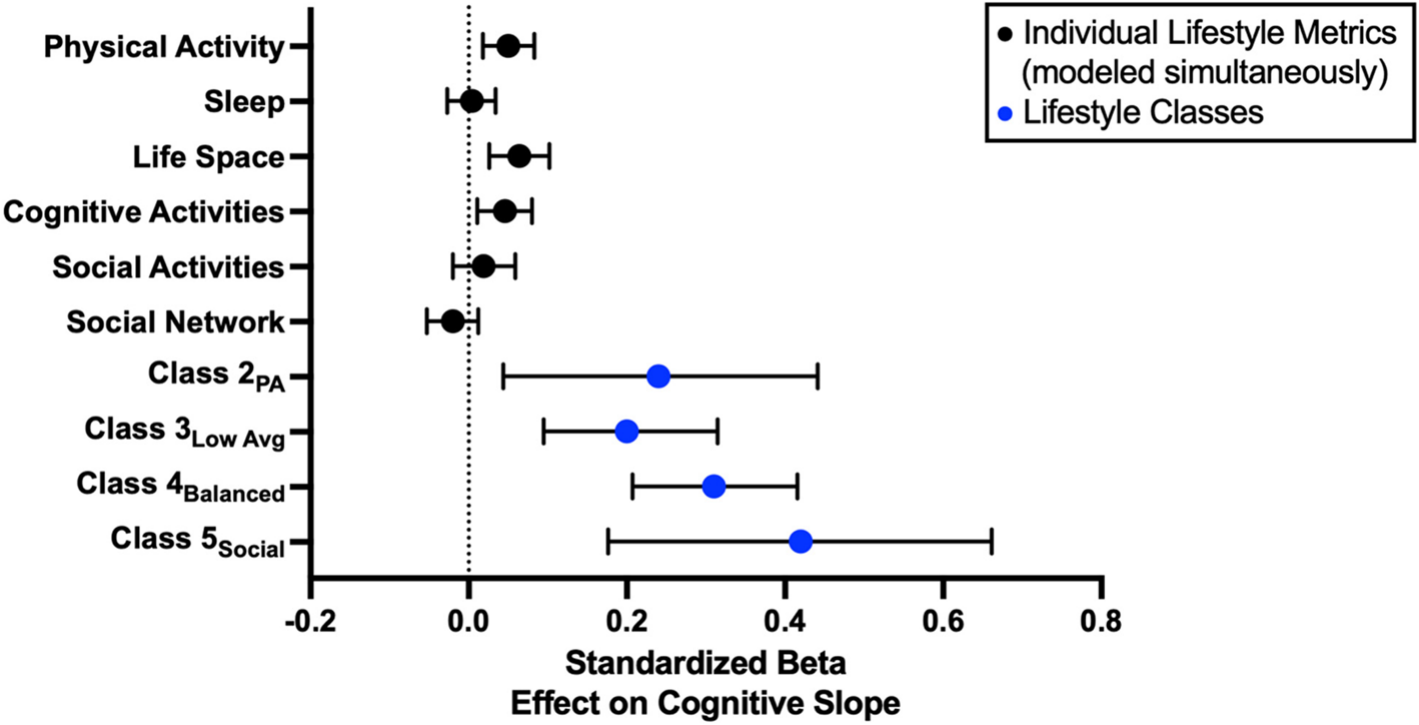
LPA-derived patterns of lifestyle factors are more predictive of global cognitive slope compared to that of each lifestyle factor individually, covarying for age, sex, and pathology. Class-specific standardized betas displayed below are the effect of each class in comparison to the reference group (i.e., class 1_Low Life Space_)

Given the significant relationships between lifestyle pattern class and cognitive trajectories, post hoc models examined the strength of association between each of the six individual lifestyle measures and cognitive trajectories to determine how the combined effects of lifestyle factors compared to any individual domain. Figure 3 displays a forest plot with standardized coefficients and 95% confidence intervals for the effects of each individual lifestyle measure and lifestyle pattern class (vs. reference group Class 1_Low Life Space_). Of the individual measures, higher physical activity, cognitive activity, and life space were statistically significantly related to slower cognitive decline over time; however, the magnitude of these relationships (*β* range = −0.020 [social network] to 0.064 [life space]) was substantially weaker than those derived from the lifestyle pattern classes, suggesting the combination of factors may be a more robust approach compared to individual behaviors explaining variance in cognitive health.

### Lifestyle classes and incident CI

**Fig. 4:**
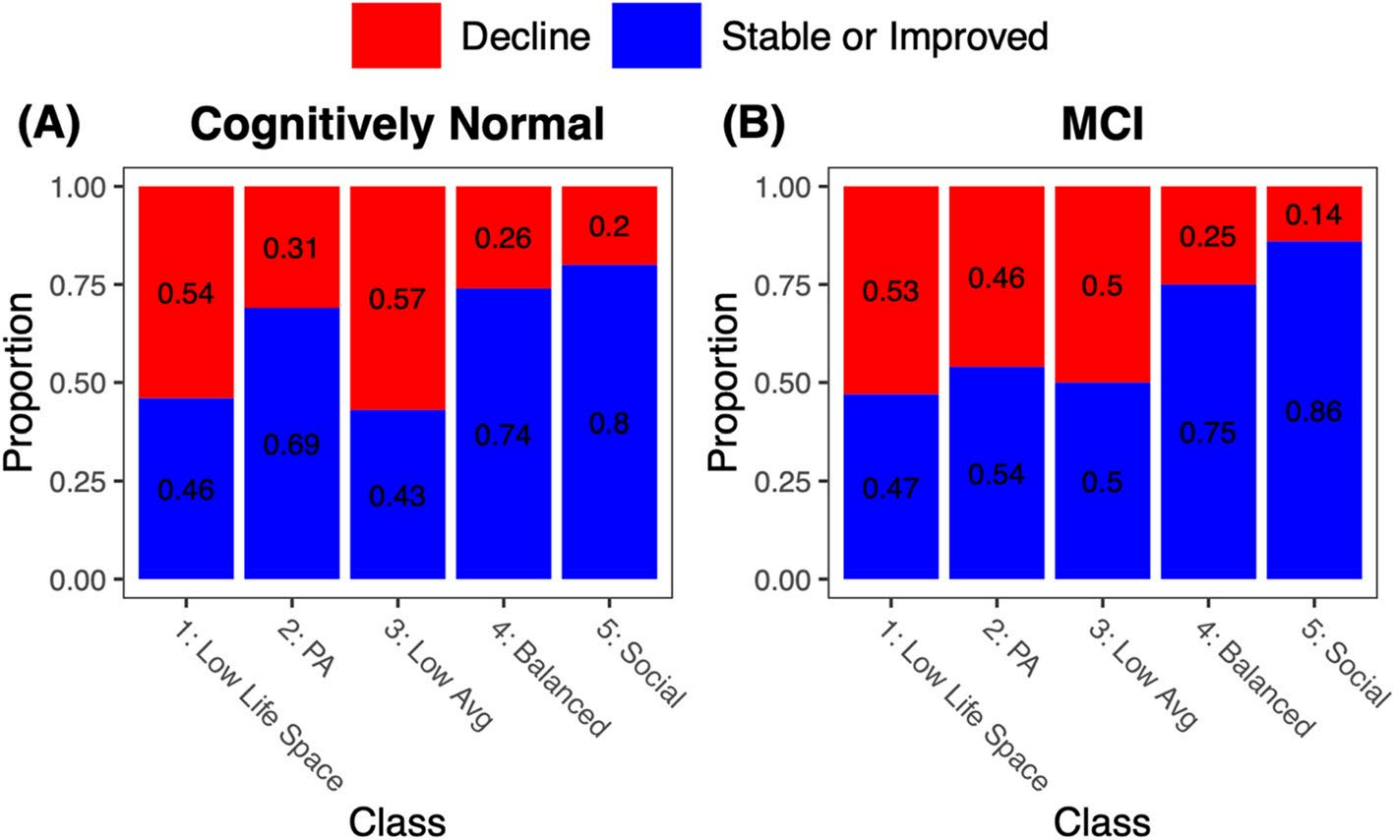
Proportion of participants who remained stable or improved versus those who clinically converted to more severe levels of cognitive impairment from first to last visit by lifestyle class. **A** shows participants who were cognitively normal at baseline, and **B** shows those who had mild cognitive impairment (MCI) at baseline

Chi-square tests examined lifestyle class differences in rates of incident CI from first to last study visit (Fig. 4). Among those who were cognitively unimpaired at baseline (*n* = 1510), class membership was significantly related to incident CI by the last study visit (*χ*^2^ = 109.4; *p* < 0.001). Highest rates of no cognitive impairment (NCI) to incident MCI/dementia were observed among those in Class 3_Low Avg_ (57%) and Class 1_Low Life Space_ (54%), with significantly lower rates of incident CI in Class 2_PA_ (31%; *p*s < 0.03), Class 4_Balanced_ (26%; *p*s < 0.001), and Class 5_Social_ (20%; *p*s < 0.001). Among those who were characterized as MCI at baseline (*n* = 549), class membership was also significantly related to incident CI by the last study visit (*χ*^2^ = 39.1; *p* < 0.001). Again, highest rates of incident CI were observed among those in Class 1_Low Life Space_ (53%) and Class 3_Low Avg_ (50%), with lower rates of incident CI in Class 2_PA_ (46%; nonsignificant, *p*s > 0.05), Class 4_Balanced_ (25%; significant, *p*s < 0.001), and Class 5_Social_ (14%; trending, *p*s = 0.058).

## Discussion

We found that discrete subgroups of older adults, defined by their pattern of engagement in multiple domains of lifestyle behaviors, exhibit differential rates of cognitive decline and incident CI. This person-specific and multimodal approach to lifestyle characterization demonstrated stronger associations with cognitive trajectories than any individual lifestyle behavior. Individuals characterized by the lowest levels of engagement in all lifestyle behaviors exhibited the steepest cognitive decline. Among the remaining lifestyle classes, attenuation of cognitive decline was observed in those with at least average levels of engagement across *all* domains, with some suggestion that social engagement may have additional positive effects on cognitive stability. Only AD and CVD burden, but not LBD or TDP-43, showed differences across lifestyle classes, such that Class 4_Balanced_ and Class 5_Social_ had the lowest AD burden, while Class 2_PA_ had the lowest CVD burden. Nonetheless, the magnitude of relationships between lifestyle classes and cognitive trajectories was robust to statistical adjustment for autopsy-defined neuropathological disease burden. Taken together, these results suggest that moderate and balanced engagement in multiple healthy lifestyle behaviors may have the most robust effect on cognitive stability, even among individuals with mild cognitive symptoms and neuropathology.

Lifestyle classes were characterized by activity-independent variation in overall engagement (e.g., Class 1_Low Life Space_ vs. Class 4_Balanced_), as well as activity-specific features (e.g., Class 2_PA_ vs. Class 5_Social_). Despite the diversity of lifestyle patterns, our most robust observation was consistent attenuation of cognitive decline among Classes 2–5 compared to Class 1_Low Life Space_. This is consistent with both observational and interventional data indicating that varied and balanced engagement in healthy lifestyle behaviors is more strongly related to cognitive stability with age than any single activity [5, 48–50]. The remaining inter-class differences in cognitive outcomes were relatively more subtle, although it is notable that the smallest magnitude of cognitive decline and lowest rate of clinical progression occurred in Class 5_Social_. The clinical relevance of social activity is supported by the 2020 Lancet Commission on dementia prevention, which estimated that infrequent social contact accounts for a similar, if not higher, population attributable fraction of dementia worldwide compared to physical inactivity ((1); 4% vs. 2%). Interestingly, social activity and social network scores were not related to cognitive slopes in analyses that modelled lifestyle factors as individual predictors, adjusting for all other factors. Thus, the strong cognitive performance among Class 5_Social_ may capture the benefits of socialization when layered upon a foundation of other healthy lifestyle engagement. In contrast, the lack of class differences in self-reported sleep quality as well as the null individual effect of sleep quality on cognitive trajectory suggests that it may not have been an important factor in this cohort; however, more objective measures of sleep may be more informative in future studies given well-studied relationships between sleep and dementia [12, 13]. Another notable lifestyle pattern was the strikingly restricted life space in Class 1_Low Life Space_ relative to other groups, consistent with prior work [51]. Life space was the only factor significantly correlated with every other lifestyle indicator, potentially reflecting reduced environmental engagement and/or mobility as a central feature of a multifaceted risk factor for cognitive decline [51].

Across pathologies, we only observed direct relationships between lifestyle patterns with cerebrovascular and Alzheimer’s disease (AD) burden. More specifically, the lifestyle class involving highest levels of physical activity showed specific neuroprotective relationships with cerebrovascular disease. These findings are consistent with well-established evidence directly linking a range of cardiometabolic lifestyle factors (e.g., exercise, body mass index) to reduced risk of cerebrovascular disease and stroke [52]. Further, lifestyle patterns involving high social connectedness or a balance of at least average or high frequency of a variety of activities and behaviors demonstrated the lowest AD burden. These results highlight the relevance of social engagement added on top of other lifestyle behaviors for cognitive health and also raise interesting hypotheses for potential brain resistance to development of AD pathology. This finding is consistent with prior in vivo studies demonstrating that among adults with NCI, those with higher social engagement had lower CSF ptau and total tau compared to isolated older adults [10]. Interestingly, LBD and TDP-43/hippocampal sclerosis did not evidence strong associations with lifestyle class membership, suggesting the relationship between lifestyle behaviors and direct risk for developing these pathologies is less prominent. Although all pathologies showed significant relationships with cognitive decline, covarying for these pathologies did not alter the degree to which lifestyle classes explained variance in cognitive slopes. These findings suggest that while lifestyle factors may contribute to some neuropathology accumulation, the majority of the biologic mechanisms linking lifestyle to cognitive health may be independent of pathology (at least as measured in this study). Taken together, these data suggest that participation in lifestyle behaviors may have high relevance for how neuropathological burden clinically manifests.

Our study is not without limitations. Although we included over 2000 older adults to derive the latent lifestyle classes, some of the class sizes were relatively small. For instance, Class 2_PA_ only included 64 individuals, suggesting that some lifestyle patterns may not be highly represented in older adults. Additionally, other than actigraphy data to capture physical activity levels, most of the other lifestyle metrics were self-report. These measures may suffer from recall bias or social desirability. Future studies leveraging technological capture of these constructs (e.g., GPS for life space, calls/texts for social activity) are warranted. Other limitations include the observational design, which cannot determine directionality in the relationship between lifestyle patterns and neuropathological or cognitive outcomes. Of note, we estimated lifestyle patterns averaged across late life to represent more “trait” level behaviors and excluded individuals with dementia at baseline to quantify lifestyle patterns before dementia onset. These methodological choices may help mitigate some issues around reverse causality.

## Conclusions

Overall, this study is among the most comprehensive assessments of lifestyle patterns in the context of cognitive aging and neuropathology. In contrast to measuring one lifestyle behavior in isolation, our data-driven approach to quantifying and characterizing multiple lifestyle patterns provides a holistic and multidimensional measurement of human behaviors that are relevant for brain health. Findings highlight the importance of multifaceted lifestyle enrichment in maintaining optimal cognition in older adulthood, even in the face of neurodegenerative pathologies. Although memory clinic providers commonly include clinical recommendations to increase physical activity, emphasis on integrating other lifestyle behaviors, particularly environmental enrichment and social activity, may be particularly relevant for bolstering brain and cognitive health in the oldest ages. Our study also provides further support for multi-domain lifestyle intervention studies to optimize cognitive health with age.

### Supplementary Information

**Additional file 1:** **Supplementary Table 1.** Linear Mixed Effects Model Results from: (A) the subset of 704 participants with autopsy data, which are identical to primary results presented in Table 2 Model 1; and (B) the entire cohort of 2059 participants.

## Data Availability

The data used in the current study are available upon request and following the completion of a data use agreement that can be completed through the Rush University Alzheimer’s Disease Center (https://www.radc.rush.edu/requests/additionalForms.htm/).

## Abbreviations

AD: Alzheimer’s disease
AIC: Akaike information criterion
BLRT: Bootstrapped likelihood ratio test
BIC: Bayesian information criterion
CI: Cognitive impairment
CAA: Cerebral amyloid angiopathy
CVD: Cerebrovascular disease
HS: Hippocampal sclerosis
LBD: Lewy body disease
LME: Linear mixed effects
LPA: Latent profile analysis
MAP: Memory and Aging Project
MCI: Mild cognitive impairment
NCI: No cognitive impairment
PA: Physical activity
PSQI: Pittsburgh Sleep Quality Index
TDP-43: Transactive response DNA-binding protein of 43 kDa
