# Supplementary material for: Multimodal lifestyle engagement patterns support cognitive stability beyond neuropathological burden
Source: Alzheimers Res Ther. 2023 Dec 18;15:221. doi: 10.1186/s13195-023-01365-9 (PMC10726589; doi:10.1186/s13195-023-01365-9)
Supplement: Supplementary file 1 — Additional file 1: Supplementary Table 1. Linear Mixed Effects Model Results from: (A) the subset of 704 participants with autopsy data, which are identical to primary results presented in Table 2 Model 1; and (B) the entire cohort of 2059 participants. [file 13195_2023_1365_MOESM1_ESM.docx]

|  | (A)  N = 704 with autopsy | | | (B)  N = 2059 entire cohort | | |
| --- | --- | --- | --- | --- | --- | --- |
|  | Std. Beta | 95% CI | *p*-value | Std. Beta | 95% CI | *p*-value |
| Baseline Age | -0.08 | -0.12, -0.05 | <0.001 | -0.10 | -0.12, -0.08 | <0.001 |
| Sex (ref: female) | -0.14 | -0.22, -0.06 | 0.001 | -0.15 | -0.2, -0.1 | <0.001 |
| Education | 0.14 | 0.11, 0.18 | <0.001 | 0.19 | 0.17, 0.21 | <0.001 |
| Total Study Visits | 0.15 | 0.12, 0.19 | <0.001 | 0.15 | 0.13, 0.17 | <0.001 |
| Time | -0.67 | -0.78, -0.57 | <0.001 | -0.69 | -0.78, -0.6 | <0.001 |
| Class 2_PA_ (ref: 1_Low Life Space_) | 0.64 | 0.29, 0.99 | <0.001 | 0.73 | 0.51, 0.94 | <0.001 |
| Class 3_Low Avg_ (ref: 1_Low Life Space_) | 0.31 | 0.13, 0.50 | 0.001 | 0.34 | 0.2, 0.49 | <0.001 |
| Class 4_Balanced_ (ref: 1_Low Life Space_) | 0.70 | 0.52, 0.87 | <0.001 | 0.84 | 0.7, 0.97 | <0.001 |
| Class 5_Social_ (ref: 1_Low Life Space_) | 0.85 | 0.43, 1.26 | <0.001 | 0.98 | 0.78, 1.19 | <0.001 |
| Time * Class 2_PA_ | 0.22 | 0.00, 0.44 | 0.049 | 0.43 | 0.29, 0.57 | <0.001 |
| Time * Class 3_Low Avg_ | 0.18 | 0.06, 0.30 | 0.004 | 0.21 | 0.12, 0.31 | <0.001 |
| Time * Class 4_Balanced_ | 0.34 | 0.22, 0.45 | <0.001 | 0.46 | 0.37, 0.55 | <0.001 |
| Time * Class 5_Social_ | 0.49 | 0.22, 0.77 | <0.001 | 0.53 | 0.39, 0.67 | <0.001 |

Supplementary Table 1. Linear Mixed Effects Model Results from: (A) the subset of 704 participants with autopsy data, which are identical to primary results presented in Table 2 Model 1; and (B) the entire cohort of 2059 participants
